# Replication and Meta-analysis of the Association between *BDNF* Val66Met Polymorphism and Cognitive Impairment in Patients Receiving Chemotherapy

**DOI:** 10.1007/s12035-018-1410-4

**Published:** 2018-10-31

**Authors:** Chia Jie Tan, Sheree Wan Ting Lim, Yi Long Toh, Terence Ng, Angie Yeo, Maung Shwe, Koon Mian Foo, Pat Chu, Amit Jain, Si-Lin Koo, Rebecca A. Dent, Raymond Chee Hui Ng, Yoon Sim Yap, Elaine H. Lim, Kiley Wei-Jen Loh, Wen Yee Chay, Guek Eng Lee, Tira Jing Ying Tan, Sok Yuen Beh, Mabel Wong, Jack Junjie Chan, Chiea Chuen Khor, Han Kiat Ho, Alexandre Chan

**Affiliations:** 1 Department of Pharmacy, Faculty of Science, National University of Singapore, Block S4, 18 Science Drive 4, Singapore, 117543 Singapore; 2 Department of Pharmacy, K.K. Women’s and Children’s Hospital, Singapore, Singapore; 3 Singapore Cord Blood Bank, Singapore, Singapore; 4 Division of Medical Oncology, National Cancer Centre, Singapore, Singapore; 5 Human Genetics, Genome Institute of Singapore, Singapore, Singapore; 6 Glaucoma Research Group, Singapore Eye Research Institute, Singapore, Singapore; 7 Department of Pharmacy, National Cancer Centre, Singapore, Singapore; 8 Duke-NUS Graduate Medical School, Singapore, Singapore

**Keywords:** Cancer-related cognitive impairment, BDNF, Genetic polymorphism, Breast cancer, Chemotherapy

## Abstract

Cancer-related cognitive impairment (CRCI) adversely affects cancer patients. We had previously demonstrated that the *BDNF* Val66Met genetic polymorphism is associated with lower odds of subjective CRCI in the multitasking and verbal ability domains among breast cancer patients receiving chemotherapy. To further assess our previous findings, we evaluated the association of *BDNF* Val66Met polymorphism with subjective and objective CRCI in a temporally separate cohort of patients and pooled findings from both the original (*n* = 145) and current (*n* = 193) cohorts in a meta-analysis. Subjective CRCI was assessed using FACT-Cog. Objective CRCI was evaluated using computerized neuropsychological tests. Genotyping was carried out using Sanger sequencing. The association of *BDNF* Val66Met genotypes and CRCI was examined with logistic regression. A fixed-effect meta-analysis was conducted using the inverse variance method. In the meta-analysis (*n* = 338), significantly lower odds of CRCI were associated with Met allele carriers based on the global FACT-Cog score (OR = 0.52, 95% CI 0.29–0.94). Furthermore, Met allele carriers were at lower odds of developing impairment in the domains of memory (OR = 0.34, 95% CI: 0.17–0.70), multitasking (OR = 0.33, 95% CI: 0.18–0.59), and verbal ability (OR = 0.46, 95% CI: 0.24–0.88). Consistent with the previous study, lower odds of subjective CRCI among patients with the *BDNF* Met allele was observed after adjusting for potential confounders in the multitasking (OR = 0.30, 95% CI: 0.14–0.67) domain. In conclusion, carriers of the *BDNF* Met allele were protected against global subjective CRCI, particularly in the domains of memory, multitasking, and verbal ability. Our findings further contribute to the understanding of CRCI pathophysiology.

**Electronic supplementary material:**

The online version of this article (10.1007/s12035-018-1410-4) contains supplementary material, which is available to authorized users.

## Introduction

Commonly known in literature as “chemobrain” or “chemofog,” subtle yet notable alterations in cognitive function are often observed in breast cancer patients receiving chemotherapy [1]. Manifesting as both patient-reported subjective complaints and objective changes detected by neuropsychological tests, cancer-related cognitive impairment (CRCI) has been reported to include memory loss, concentration deficit, and the decreased ability to multitask [2]. Evidence has shown that CRCI negatively affects the quality of life of cancer patients and the ability to cope with demands in their daily lives [3]. As its etiology is not yet fully understood, CRCI remains a subject of significant research. Ongoing work has suggested possible factors that may influence the risk of CRCI, such as pro-inflammatory cytokines, psychosocial determinants including anxiety and fatigue, and numerous genetic markers [4–6]. Among candidate genes that have been investigated are *COMT*, *APOE*, and *BDNF* [6, 7].

The *BDNF* gene expresses brain-derived neurotrophic factor (BDNF), which is a neurotrophic factor vital for neuronal survival, growth, and neural circuit maintenance [8]. The *BDNF* Val66Met single nucleotide polymorphism (SNP), which leads to substitution of valine with methionine at codon 66, is a functional polymorphism widely studied in neurological conditions such as schizophrenia and Parkinson’s disease [9, 10]. Our research group has discovered that the *BDNF* Val66Met polymorphism is associated with a lower risk of developing self-perceived CRCI in breast cancer patients [7], where carriers of the Met allele had lower odds of reporting subjective CRCI in the cognitive domains of verbal ability (OR = 0.34, 95% CI = 0.12–0.90) and multitasking (OR = 0.37, 95% CI = 0.15–0.91). However, other studies have suggested that carrying the Met allele may be associated with poorer perseveration, verbal memory abilities, and task switching [11]. Therefore, whether carriers of the Met allele are truly protected against cognitive decline remains controversial, implying that the *BDNF* Val66Met polymorphism may contribute to varying cognitive function [11].

As false positives are commonly observed in genetic association studies [12], further replication attempts are required to confirm associations that were initially observed, in order to provide stronger evidence on the impact of genetic determinants on CRCI. A deeper understanding of these genetic factors will allow the identification of cancer patients at a higher risk of CRCI for potential interventions. Therefore, in this study, we aim to evaluate the association of *BDNF* Val66Met polymorphism with subjective and objective CRCI in a temporally separate cohort of patients and pool findings from both the original and current cohorts in a meta-analysis.

## Methods

### Study Design

This was a multicenter, prospective cohort study conducted at three ambulatory cancer centers between February 2014 and December 2017 in Singapore. This study was approved by SingHealth Institutional Review Board (CIRB2014/754/B) and written informed consent was obtained from all patients.

### Study Population

Eligible participants must fulfill the following inclusion criteria: (i) at least 21 years old, (ii) diagnosed with stages I to III breast cancer, (iii) scheduled to receive chemotherapy, (iv) has no prior history of chemotherapy and/or radiotherapy, (v) able to read and understand either English or Mandarin, and (vi) has Eastern Cooperative Oncology Group (ECOG) performance status score of 0 or 1.

Patients were excluded from the study if they were (i) incapable of providing verbal/written consent or (ii) diagnosed with neuropsychiatric disorders and/or brain metastasis that might result in poor cognitive function.

### Study Procedures

Upon recruitment, demographic data and clinical information of participants were collected via patient interviews and from electronic medical records. Participants were prospectively evaluated at three time points: before start of chemotherapy (T1), 6 weeks after start of chemotherapy (T2), and 12 weeks after start of chemotherapy (T3). At each time point, participants completed assessments of both subjective and objective CRCI. In addition, health-related quality of life, anxiety, and fatigue were assessed using self-administered questionnaires. English and Chinese versions of each study tool were available. All assessments took approximately 45 min to complete and were conducted by trained interviewers.

### Assessment of Subjective Cognitive Impairment

The Functional Assessment of Cancer Therapy–Cognitive Function (FACT-Cog) version 3 was used to evaluate patients’ self-perceived CRCI within the past 7 days [13]. FACT-Cog comprises 37 items in 6 domains of cognitive disturbances, which are mental acuity, concentration, memory, verbal ability, functional interference, and multi-tasking ability. Each item is evaluated on a 5-point Likert scale. Both English and Chinese versions of FACT-Cog have been validated and demonstrated satisfactory psychometric properties [14].

Subjective CRCI is defined as a reduction of at least 10.6 points in the FACT-Cog total score at T2 or T3 compared to baseline based on a previously determined minimal clinically important difference (MCID). Decline in a particular cognitive domain is defined as a reduction of at least 15% from a participant’s baseline score at T2 or T3 [15].

### Assessment of Objective Cognitive Impairment

Objective cognitive assessment was carried out using the Cambridge Neuropsychological Test Automated Battery (CANTAB), a language-independent neuropsychological testing research software. In this study, the CANTAB test battery contained five tests: reaction time (RTI), paired associates learning (PAL), spatial working memory (SWM), attention switching task (AST), and rapid visual information processing (RVP) that assessed response speed, learning and memory, working memory, multitasking, and sustained attention, respectively, yielding a total of nine measures. The direction of one measure, *A′*, was reversed so higher scores indicate poorer cognitive performance for all measures. These tests have been validated and have shown sensitivity to capturing alterations in neuropsychological performance [16–18].

Reliable change indices (RCI) were computed to reflect cognitive changes in participants. RCI were obtained by subtracting CANTAB scores at T2 or T3 from baseline scores, adjusting for practice effects and dividing by the standard error of difference. Practice effects and standard error of difference were estimated from a control population using similar testing intervals. Objective cognitive decline is defined as an RCI of less than − 2 at either T2 or T3.

### Assessment of Fatigue

Fatigue was evaluated with the Brief Fatigue Inventory (BFI) [19]. BFI measures the severity of fatigue and the impact of fatigue on daily functioning in the past 24 h on a numerical scale of 0 to 10. Six aspects of daily functioning were assessed: general activity, mood, walking ability, normal work, relations with other people, and enjoyment of life. A higher score indicates greater level of fatigue.

### Assessment of Anxiety

The Beck Anxiety Inventory (BAI) was employed to measure anxiety in participants [20, 21]. BAI is a validated questionnaire consisting of 21 items describing subjective, somatic, or panic-related symptoms of anxiety on a scale of 0 to 3. A higher total score indicates greater level of anxiety.

### Assessment of Insomnia

The European Organization for Research and Treatment of Cancer Quality of Life Questionnaire (EORTC QLQ-C30) assesses health-related quality of life (HRQoL). In this study, we focused on the single-item scale rating insomnia, which is measured on a 4-point Likert scale. A higher score indicates increased severity of insomnia. Both English and Chinese versions of QLQ-C30 have been validated for use in cancer patients in Singapore [22, 23].

### Genotyping

At baseline, a 10-ml blood sample was collected from participants in an ethylene diamine tetraacetic acid (EDTA) tube and centrifuged at 2500 rpm for 10 min within 40 min of collection. The buffy coat was extracted and at stored at − 80 °C until analysis.

Using QIAamp DNA Blood Mini Kit (QIAGEN), genomic DNA from the buffy coat was isolated. The region with the *BDNF* Val66Met polymorphism was amplified via polymerase chain reaction (PCR) using the following specific and optimized primers: 5′-GGACTCTGGAGAGCGTGAA-3′ (forward) and 5′-CGTGTACAAGTCTGCGTCCT-3′ (reverse). Genotyping of the PCR products was subsequently conducted by AITbiotech employing automated Sanger sequencing with a 3730xl DNA Analyzer (Applied Biosystems). AITbiotech was blinded to clinical outcomes of participants. To ensure quality control, genotyping was done for both the forward and reverse DNA strands.

### Statistical Analysis

All statistical analyses were conducted with STATA Version 15 (StataCorp 2017). Descriptive statistics were used to summarize demographics and clinical characteristics of participants. Deviation of genotypes from Hardy-Weinberg equilibrium was assessed using chi-squared test with one degree of freedom. Evaluation of the associations between the *BDNF* Val66Met polymorphism and CRCI was done using logistic regression assuming a dominant model. Potential confounders age, race, menopausal status, chemotherapy regimens, years of education, and additionally for subjective CRCI, anxiety, depression, and insomnia were adjusted for [24, 25]. Sensitivity analyses was performed assuming a general genetic model with each genotype classified as a distinct class. To examine the relationship between anxiety and fatigue with BDNF genotype, linear mixed-effect models were employed with the presence of Met allele and time incorporated as fixed effects and intercepts varied as a random effect by each subject. To combine findings from both the original and current cohort, adjusted odds ratios from both studies were pooled in a fixed-effect meta-analysis using the inverse variance method. All statistical tests were two-sided, and *p* values less than 0.05 were considered statistically significant.

### Sample Size Calculation

Sample size calculation was performed using Quanto 1.2.4. In our original study, statistically significant association of CRCI with *BDNF* genotype was observed for the cognitive domains of verbal ability (OR = 0.34) and multitasking (OR = 0.37) [7]. The latter, which yielded a smaller effect size, was used for sample size estimation in this study. Based on an expected allelic frequency of 0.5 in a dominant model and predicted prevalence of impairment at 0.3 [7], a total of 167 participants is required to yield statistical power of 80% and type 1 error of 5%. Anticipating an attrition rate of 20%, a final sample size of 209 was targeted.

## Results

### Patient Characteristics

**Fig. 1:**
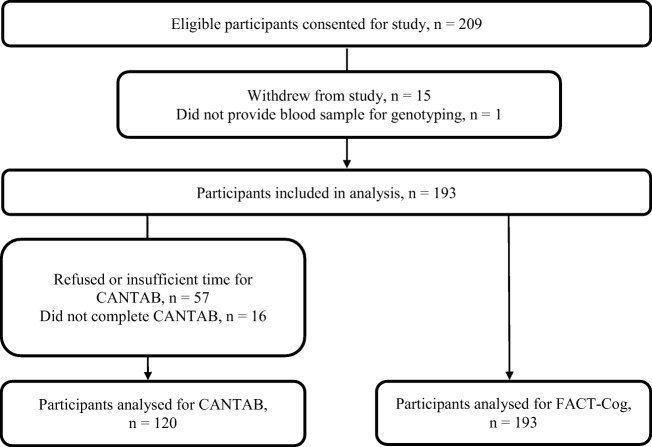
Study flow diagram

A total of 209 patients were recruited. However, 15 participants withdrew from the study (2 patients refused chemotherapy and 13 declined to complete study procedures) and 1 patient did not provide blood samples for genotyping. Therefore, 193 participants were included in the final analysis (Fig. 1). The demographic characteristics of patients who dropped out and those who remained in the study did not differ significantly. The mean (±SD) age of participants was 51.9 ± 8.9 years old. Majority of the participants were of Chinese ethnicity (79.8%) and had at least high school education (84.5%). More than half received radiotherapy (66.3%), underwent mastectomy (63.2%), and completed anthracycline-based chemotherapy (64.8%). Demographic and clinical characteristics of participants in both the current and original cohorts are comparable (Table 1).

**Table 1:** Comparison of demographic and clinical characteristics of participants in the current cohort and original cohort

	Current cohort	Original cohort
	*n* = 193	*n* = 145
Demographic characteristics		
Age in years, mean (SD)	51.9 (8.9)	50.8 (8.8)
Ethnicity, *n* (%)		
Chinese	154 (79.8)	119 (82.1)
Malay	19 (9.8)	15 (10.3)
Indian	13 (6.7)	7 (4.8)
Others	7 (3.6)	4 (2.8)
Education level, *n* (%)		
Primary school	29 (15.0)	22 (15.2)
High school	90 (46.6)	70 (48.3)
Pre-university	35 (18.1)	29 (20.0)
Graduate/postgraduate	38 (19.7)	24 (16.6)
Unknown	1 (0.5)	0 (0.0)
Menopausal status, *n* (%)		
Premenopausal	95 (49.2)	74 (51.0)
Postmenopausal	98 (50.8)	71 (49.0)
Clinical characteristics		
Cancer staging, *n* (%)		
Stage I	27 (14.0)	32 (22.1)
Stage II	127 (65.8)	71 (49.7)
Stage III	39 (20.2)	41 (28.3)
Radiotherapy, *n* (%)	128 (66.3)	Not reported
Surgery, *n* (%)		Not reported
Lumpectomy	71 (36.8)	
Mastectomy	122 (63.2)	
Chemotherapy, *n* (%)		
Anthracycline-based	125 (64.8)	94 (64.8)
Non anthracycline-based	68 (35.2)	51 (35.2)
Behavioral symptoms		
Baseline fatigue, mean (SD)	1.6 (1.9)	1.6 (1.7)
Baseline anxiety, mean (SD)	6.9 (7.2)	6.7 (6.1)
Baseline insomnia, mean (SD)	22.8 (26.8)	23.1 (26.9)

### Genotype and Allele Frequencies

All participants included in the final analysis were successfully genotyped for the *BDNF* Val66Met polymorphism. Val/Val and Met/Met homozygous genotypes accounted for 26.9% and 20.7% of the observed genotypes, respectively, while the remaining 52.3% comprised the heterozygous genotype. Val and Met allele frequencies were approximately equivalent. No deviation from Hardy-Weinberg equilibrium was detected whether allele frequencies were pooled or stratified by ethnicity (Table 2).

**Table 2:** Genotype and allele frequencies of participants (n = 193)

Genotype/allele	Ethnic subpopulation, *n* (%)				Pooled, *n* (%)
	Chinese	Malay	Indian	Others^b^	
Total	154	19	13	7	193
Genotype					
GG (Val/Val)	35 (22.7)	10 (52.6)	4 (30.8)	3 (42.9)	52 (26.9)
GA (Val/Met)	84 (54.6)	6 (31.6)	7 (53.8)	4 (57.1)	101 (52.3)
AA (Met/Met)	35 (22.7)	3 (15.8)	2 (15.4)	0 (0.0)	40 (20.7)
Allele					
G (Val) allele	154 (50.0)	26 (68.4)	15 (57.7)	10 (71.4)	205 (53.1)
A (Met) allele	154 (50.0)	12 (31.6)	11 (42.3)	4 (28.6)	181 (46.9)
*p* value^a^	0.26	0.24	0.71	0.29	0.48

^a^p values of Chi-square tests to assess deviation from Hardy-Weinberg equilibrium

^b^“Others” include Sri Lankan, Filipino, and Burmese

### Prevalence of Subjective and Objective Cognitive Impairment

A total of 193 participants completed FACT-Cog evaluation and 60 patients (31.1%) reported subjective CRCI (Table 3). Among specific cognitive domain, the highest proportion of participants reporting cognitive decline was observed in the mental acuity (28.5%) domains, followed by concentration (28.0%), multi-tasking (25.9%), verbal ability (20.2%), functional interference (19.2%), and memory (17.6%).

**Table 3:** Proportion of participants with CRCI

	Proportion of participants, *n* (%)
Subjective CRCI (*n* = 193)	
Summation score	60 (31.1)
Cognitive domains	
Memory	34 (17.6)
Verbal ability	39 (20.2)
Concentration	54 (28.0)
Mental acuity	55 (28.5)
Functional interference	37 (19.2)
Multitasking	50 (25.9)
Decline in at least 1 domain	88 (45.6)
Objective CRCI (*n* = 120)	
Individual test measures	
RTI – Five choice reaction time	17 (14.2)
PAL – Total error (adjusted)	15 (12.5)
SWM – Between errors	5 (4.2)
SWM – Strategy	15 (12.5)
AST – Switching cost	3 (2.5)
AST – Congruency cost	3 (2.5)
AST – Reaction latency	8 (6.7)
RVP – *A′*	8 (6.8)
RVP – Latency	20 (16.7)
Cognitive domains	
Response speed	17 (14.2)
Learning and memory	15 (12.5)
Working memory	17 (14.2)
Multitasking	10 (8.3)
Sustained attention	28 (23.3)
Decline in at least 1 domain	59 (49.2)
Number of domains	
1	40 (33.3)
2	11 (9.2)
3	7 (5.8)
4	1 (0.83)

A total of 120 participants completed CANTAB assessments. Participants who completed CANTAB assessments were younger, more likely to be pre-menopausal and attained higher education levels than those who did not. However, there was no difference in baseline anxiety and fatigue levels, proportion reporting subjective CRCI and *BDNF* Val66Met genotypic distribution between the two groups of patients (Supplementary Table S1). A total of 59 individuals, representing nearly half of the patients (49.2%) experienced decline in at least one cognitive domain (Table 3). The highest proportion of patients with cognitive decline was reported in the domain of sustained attention (23.3%), followed by response speed (14.2%), working memory (14.2%), learning and memory (12.5%), and multitasking (8.3%).

### Association of BDNF Genotypes with Cognitive Impairment

After adjusting for potential confounders including anxiety and fatigue, Met allele carriers showed a consistent trend of decreasing odds of subjective CRCI across all domains; however, statistical significance was only observed in memory (OR = 0.24, 95% CI = 0.09–0.61); multitasking (OR = 0.30, 95% CI = 0.14–0.67); and mental acuity (OR = 0.46, 95% CI = 0.21–0.99). Apart for the mental acuity domain (unadjusted OR = 0.53, 95% CI = 0.27–1.04), adjusting for potential confounders did not alter the significance of association (Table 4). In contrast, no significant associations were detected in all cognitive domains investigated for objective CRCI in both adjusted and unadjusted analysis (Table 4). Analysis performed assuming a general genetic model yielded results with similar trends (Supplementary Table S2).

**Table 4:** Association of carrying *BDNF* Met allele with CRCI

Variable	Unadjusted analysis		Adjusted analysis	
	OR	*p* value	OR	*p* value
Subjective cognitive impairment				
	(*n* = 193)		(*n* = 192)^a^	
Total score	0.63 (0.32–1.24)	0.18	0.62 (0.29–1.30)	0.21
Memory	0.45 (0.21–0.97)	0.04^b^	0.24 (0.09–0.61)	0.003^b^
Multitasking	0.43 (0.22–0.86)	0.02^b^	0.30 (0.14–0.67)	0.003^b^
Verbal ability	0.58 (0.28–1.24)	0.16	0.57 (0.24–1.38)	0.22
Concentration	0.98 (0.48–1.99)	0.96	0.86 (0.38–1.90)	0.70
Mental acuity	0.53 (0.27–1.04)	0.07	0.46 (0.21–0.99)	0.047^b^
Functional interference	0.87 (0.40–1.93)	0.74	0.69 (0.27–1.75)	0.44
Objective cognitive impairment				
	(*n* = 120)		(*n* = 119)^a^	
Response speed	2.01 (0.54–7.49)	0.30	3.02 (0.69–13.26)	0.14
Learning and memory	1.10 (0.32–3.73)	0.88	1.58 (0.36–6.87)	0.54
Working memory	1.34 (0.40–4.43)	0.64	1.32 (0.35–4.94)	0.68
Multitasking	0.36 (0.10–1.33)	0.12	0.32 (0.06–1.70)	0.18
Sustained attention	2.12 (0.73–6.13)	0.17	3.02 (0.88–10.35)	0.08

^a^Insufficient covariate data for 1 participant

^b^*p* < 0.05

### Trajectory of Fatigue and Anxiety and Association with BDNF Genotypes

Mean scores of BFI and BAI, indicating fatigue and anxiety, respectively, showed an increasing trend over time (Table 5). Baseline fatigue and anxiety were also shown to be significant predictors of subjective CRCI in univariate analysis (Supplementary Table S3); however, further analysis showed that anxiety and fatigue levels over time were not associated with *BDNF* Val66Met polymorphism.

**Table 5:** Association of *BDNF* Met allele with fatigue and anxiety over time

	Mean scores (SD)			β	*p* value
	T1	T2	T3		
Fatigue (BFI)					
All participants	1.64 (1.89)	1.91 (2.03)	2.23 (2.05)		
Met allele carrier					
No	1.39 (1.56)	2.15 (2.08)	2.40 (2.04)	Reference	
Yes	1.74 (1.99)	1.81 (2.01)	2.17 (2.05)	− 0.07	0.77
Anxiety (BAI)					
All participants	6.86 (7.23)	8.22 (8.55)	8.55 (7.66)		
Met allele carrier					
No	7.00 (8.79)	8.87 (8.52)	9.40 (7.42)	Reference	
Yes	6.82 (6.60)	7.98 (8.58)	8.23 (7.74)	− 0.75	0.48

### Meta-Analysis of Association between BDNF Genotypes and Subjective CRCI

Meta-analysis of odds ratios from the original (*n* = 145) and current (*n* = 193) cohorts showed comparable trends with consistent directions of association. Significantly lower odds of CRCI were associated with Met allele carriers in the domains of memory (OR = 0.34, 95% CI = 0.17–0.70); multitasking (OR = 0.33, 95% CI = 0.18–0.59); and verbal ability (OR = 0.46, 95% CI = 0.24–0.88) (Table 6). In addition, the pooled odds ratio of subjective CRCI based on total FACT-Cog score was also lower in Met allele carriers (OR = 0.52, 95% CI = 0.29–0.94) (Table 6). No significant heterogeneity was detected between the two studies for all domains (*I*^2^ = 0–34%).

**Table 6:** Pooled odds ratios of CRCI among patients carrying BDNF Met allele (Val/Met or Met/Met) compared to Val/Val genotype

Domain	Cohort	OR (95% CI)	Weight	Pooled OR (95% CI)	*p* value	*I*^2^ (%)
Summation	Previous	0.40 (0.16–1.04)	39.1	0.52 (0.29–0.94)	0.03^a^	0
	Current	0.62 (0.29–1.30)	60.9			
Memory	Previous	0.53 (0.19–1.53)	45.7	0.34 (0.17–0.70)	0.003^a^	17
	Current	0.24 (0.09–0.61)	54.3			
Multitasking	Previous	0.37 (0.15–0.91)	43.0	0.33 (0.18–0.59)	< 0.001^a^	0
	Current	0.30 (0.14–0.67)	57.0			
Verbal ability	Previous	0.34 (0.12–0.90)	43.0	0.46 (0.24–0.88)	0.02^a^	0
	Current	0.57 (0.24–1.38)	57.0			
Concentration	Previous	0.61 (0.23–1.59)	40.9	0.75 (0.40–1.39)	0.36	0
	Current	0.86 (0.38–1.90)	59.1			
Mental acuity	Previous	1.03 (0.37–2.86)	36.5	0.62 (0.33–1.15)	0.13	34
	Current	0.46 (0.21–0.99)	63.5			
Functional interference	Previous	0.38 (0.13–1.14)	42.6	0.54 (0.26–1.09)	0.08	0
	Current	0.69 (0.27–1.75)	57.4			

^a^*p* < 0.05

## Discussion

Findings in this well-powered study and pooled results from both the original and current studies show that carriers of the *BDNF* Met allele is associated with a trend towards lower odds of reporting self-perceived CRCI across different domains. This replicates the protective effect of *BDNF* Val66Met on subjective CRCI we have observed in our previous work. Consistent with our previous report, *BDNF*Val66Met was not associated with objective CRCI. Further meta-analysis of the original and the current cohort have also uncovered the protective effect between *BDNF*Val66Met and global subjective CRCI. This is a novel finding that has not been reported in the literature.

To date, the only other studies investigating the effect of *BDNF* Val66Met polymorphism on self-perceived cognitive function have been carried out in healthy individuals and did not report lower odds of subjective CRCI among *BDNF* Met carriers [26, 27]. Therefore, we postulate that the protective effect of *BDNF* Val66Met polymorphism on cognitive impairment is conditional on the presence of active malignancy or ongoing cancer treatment, both which have been hypothesized as possible causes of CRCI [25]. This discrepancy may be explained by animal studies where the expression and release of BDNF have been shown to be heavily dependent on other physiological elements, such as stress and inflammation, which are elevated in cancer patients undergoing treatment [28]. It has also been demonstrated that as cancer patients undergo chemotherapy, changes in plasma BDNF levels differ between *BDNF* genotypes [29]. These observations indicate that the effect of genetic polymorphisms may be mediated by downstream mechanisms that vary in different disease states. To further elucidate the links between *BDNF* Val66Met polymorphism and CRCI, it will be useful to investigate and compare differences in gene and protein expression between *BDNF* genotypes in both healthy and cancer patient populations. This will not only enhance our understanding of how genetic factors influence the development of CRCI but also provide insights to the pathophysiology of CRCI.

Our earlier study showed a significant association of carrying the *BDNF* Met allele with decreased odds of self-perceived CRCI in the FACT-Cog domains of multitasking and verbal ability [7]. In this study, we were able to replicate our previous findings in multitasking ability but not in verbal ability although demographic and clinical characteristics of both the original and current cohorts were comparable. While similar directions of association were observed, the effect size of carrying the Met allele was smaller and did not achieve statistical significance (OR in this study = 0.57, OR in original study = 0.34). A possible explanation for this non-replication could be genetic heterogeneity, where impairment in the verbal ability domain may not be specific to the *BDNF* gene but also associated with other genes not covered in our studies. Another possibility is the phenomenon described as “winner’s curse” where effect sizes are often found to be overestimated in initial genetic association studies [12, 30]. Replication attempts subsequently yield smaller effect sizes and as a result, studies are underpowered to detect a significant impact of genetic polymorphisms. In contrast, our meta-analysis has detected a significant association between the genetic polymorphism and global subjective CRCI, which was not reported in the previous study. The original cohort was not adequately powered to evaluate the effect size, and this limitation has been overcome by the combined analysis of both cohorts.

In contrast to subjective CRCI, we did not detect any significant association between *BDNF* Val66Met polymorphism and objective CRCI, a trend which is consistent with findings from our previous work as well as other studies in breast and brain tumor patients [7, 31, 32]. The lack of agreement between trends and predictors of objective and subjective CRCI is counter-intuitive but has been commonly reported in literature [33]. Subjective reports of cognitive function are more reflective of the ability to complete daily activities, which require the coordination of different cognitive skills, some which may not have been measured by specific neuropsychological tests used to assess objective cognitive function. While these tests are widely acknowledged as the gold standard to assess cognitive function, the importance of subjective cognitive reports should not be dismissed as they portray the impact of impaired cognition on the daily functioning of patients. Future work in this area should emulate our study, incorporating both objective and subjective measures of cognitive function as both outcomes hold equal importance and are consistently shown to be poorly correlated with each other.

Past research has suggested that subjective cognitive impairment is closely linked to other chemotherapy-related symptoms such as anxiety, depression, and fatigue hence may be more indicative of emotional distress rather than compromised cognitive function [1]. One may therefore speculate that *BDNF* Val66Met polymorphism may be protective against these accompanying symptoms rather than CRCI, explaining the lack of agreement between the association of *BDNF* polymorphism with objective and subjective CRCI observed in our study. Nevertheless, anxiety and fatigue levels have been adjusted for in our analysis. Although we did not observe any significant associations in this study, other genetic association studies have also shown that unlike subjective CRCI, anxiety, fatigue, and depression are not ameliorated but worsened among *BDNF* Met carriers [34, 35]. Considering the combination of these facts, we are confident that our observations are due to true associations of *BDNF* Val66Met polymorphism with reduced odds of subjective cognitive decline rather than the confounding effects of fatigue or other psychosocial factors.

In genetic association studies, replication attempts are crucial to confirm initial findings. Past studies have suggested several genetic polymorphisms as possible predictors of CRCI [36, 37] but to the best of our knowledge, none have never been successfully replicated. For example, the effect of *APOE* ε4 allele was first observed in breast cancer and lymphoma survivors, but similar associations have not been replicated in similar patient populations and cognitive domains [32, 38, 39]. The association of *COMT* Val158Met with cognitive impairment in breast cancer survivors has only been successfully replicated in patients with brain tumors [31]. Thus, this study is essential as it replicates observed associations of similar direction and strength in the same cognitive domains and study population.

A limitation of our study is that a different neuropsychological test battery, CANTAB, was employed to assess objective cognitive function as the Headminder system used in the original study was no longer commercially available. To reduce any potential discrepancy between Headminder and CANTAB, we ensured that all neuropsychological tests used were validated for similar cognitive domains. Furthermore, the RCI calculated to measure cognitive changes is standardized by dividing differences in test scores at two separate time points by the standard error of measurement. This ensures that score changes in both studies are comparable although different tests were utilized. Nevertheless, we acknowledge that the use of different cognitive assessment tools makes comparison between studies challenging. Furthermore, meta-analysis of findings on objective CRCI from both studies could not be performed as different neuropsychological tools were employed. In genetic association studies where replication attempts and meta-analyses are highly encouraged to increase the effective sample size for a more robust estimate of the genetic effect, it is imperative that similar tools to measure cognitive ability are used across different studies. It should also be noted that a proportion of participants did not complete CANTAB assessments. Given that patients who failed to complete CANTAB assessments were older, more likely to be post-menopausal, and received less education, the prevalence of objective cognitive decline may have been underestimated in this study. Nevertheless, we believe that this is unlikely to influence the lack of association that we observed between *BDNF* genotype and objective CRCI, as age, education level, and menopausal status were controlled for in our regression analysis. Furthermore, the *BDNF* Val66Met genotypic distribution between participants who had and had not completed CANTAB assessment were also found to be comparable.

In conclusion, carriers of the *BDNF* Met allele were protected against global subjective CRCI, particularly in the domains of memory, multitasking, and verbal ability. Similar trends towards reduced odds of subjective CRCI in all other cognitive domains were also observed in both original and current cohorts. We have also confirmed that no association could be detected between *BDNF* Val66Met polymorphism and objective cognitive function. As cancer- and treatment-related toxicities such as CRCI have been shown to have a devastating impact on cancer survivors, prediction models to estimate the risk of these toxicities should be established and tested for clinical use, so that survivors at risk can be targeted at an earlier stage for interventional measures to improve their daily functioning and quality of life. Genetic markers, such as *BDNF* Val66Met, should be incorporated in these prediction models once they have been validated in other cancer populations. Augmented with more findings from gene and protein expression studies, this work will contribute to current knowledge on the biochemical pathways that are involved with the development of CRCI. This allows us to identify potential drug targets, which can be further screened for candidates of pharmacological interventions to attenuate the negative impact of CRCI among cancer patients.

## Electronic supplementary material

ESM 1 (DOCX 38 kb)
