# Supplementary material for: Replication and Meta-analysis of the Association between BDNF Val66Met Polymorphism and Cognitive Impairment in Patients Receiving Chemotherapy
Source: Mol Neurobiol. 2018 Oct 31;56(7):4741–50. doi: 10.1007/s12035-018-1410-4 (PMC6647505; doi:10.1007/s12035-018-1410-4)
Supplement: Supplementary file 1 — (DOCX 38 kb) [file 12035_2018_1410_MOESM1_ESM.docx]

**Supplementary tables**

Supplementary Table S1: Characteristics of participants who completed and did not complete CANTAB assessments

|  | Participants who completed CANTAB | |  |
| --- | --- | --- | --- |
|  | No | Yes | p-value |
|  | n = 73 | n = 120 |  |
| **Demographic characteristics** | | |  |
| Age in years, mean (SD) | 53.7 (9.4) | 50.8 (8.5) | 0.03 |
| Ethnicity, n (%) |  |  |  |
| Chinese | 57 (78.1) | 97 (80.8) | 0.09 |
| Malay | 9 (12.3) | 10 (8.3) |  |
| Indian | 7 (9.6) | 6 (5.0) |  |
| Others | 0 (0.0) | 7 (5.8) |  |
| Education level, n (%) |  |  |  |
| Primary school | 17 (23.3) | 12 (10.0) | 0.03 |
| High school | 36 (49.3) | 54 (45.0) |  |
| Pre-university | 9 (12.3) | 26 (21.7) |  |
| Postgraduate | 11 (15.1) | 27 (22.5) |  |
| Unknown | 0 (0.0) | 1 (0.8) |  |
| Menopausal status, n (%) |  |  |  |
| Premenopausal | 28 (38.4%) | 67 (55.8%) | 0.02 |
| Postmenopausal | 45 (61.6%) | 53 (44.2%) |  |
| **Clinical characteristics** |  |  |  |
| Cancer staging, n (%) |  |  |  |
| Stage I | 8 (11.0) | 19 (15.8) | 0.52 |
| Stage II | 48 (65.8) | 79 (65.8) |  |
| Stage III | 17 (23.3) | 22 (18.3) |  |
| Radiotherapy, n (%) | 46 (63.0) | 82 (68.3) | 0.45 |
| Surgery, n (%) |  |  |  |
| Lumpectomy | 24 (32.9) | 47 (39.2) | 0.38 |
| Mastectomy | 49 (67.1) | 73 (60.8) |  |
| Chemotherapy, n (%) |  |  |  |
| Anthracycline-based | 48 (65.8) | 77 (64.2) | 0.82 |
| Non anthracycline-based | 25 (34.2) | 43 (35.8) |  |
| **Behavioral symptoms** |  |  |  |
| Baseline fatigue, mean (SD) | 1.4 (1.8) | 1.8 (1.9) | 0.10 |
| Baseline anxiety, mean (SD) | 6.0 (4.7) | 7.4 (8.4) | 0.21 |
| Baseline insomnia, mean (SD) | 19.2 (22.9) | 25.0 (28.7) | 0.14 |
| Proportion with subjective CRCI, n (%) | 22 (30.1) | 38 (31.7) | 0.82 |
| **Genotypic distribution** |  |  |  |
| GG (Val/Val), n (%) | 18 (24.7) | 34 (28.3) | 0.85 |
| AG (Val/Met), n (%) | 39 (53.4) | 62 (51.7) |  |
| AA (Met/Met), n (%) | 16 (21.9) | 24 (20.0) |  |

Supplementary Table S2 Association of BDNF genotype with cognitive decline assuming a general genetic model

| Variable | Impairment | | | Unadjusted analysis | | Adjusted analysis | |
| --- | --- | --- | --- | --- | --- | --- | --- |
|  | No, n (%) | Yes, n (%) | | OR | p-value | OR | p-value |
| **Subjective cognitive function** | | | | **(n = 193)** | | **(n = 192)*** | |
| Total score | | | | | | | |
| Genotype |  |  |  | |  |  |  |
| Val/Val | 32 (24.1) | 20 (33.3) | Baseline | | | Baseline | |
| Val/Met | 74 (55.6) | 27 (45.0) | 0.58 (0.29-1.19) | | 0.14 | 0.55 (0.25-1.21) | 0.14 |
| Met/Met | 27 (20.3) | 13 (21.7) | 0.77 (0.32-1.83) | | 0.56 | 0.83 (0.31-2.17) | 0.70 |
| Memory | | | | | | | |
| Genotype |  |  |  | |  |  |  |
| Val/Val | 38 (23.9) | 14 (41.2) | Baseline | | | Baseline | |
| Val/Met | 88 (55.4) | 13 (38.2) | 0.40 (0.17-0.93) | | 0.03** | 0.23 (0.09-0.61) | 0.003** |
| Met/Met | 33 (20.8) | 7 (20.6) | 0.58 (0.21-1.60) | | 0.29 | 0.27 (0.08-1.00) | 0.05 |
| Multitasking | | | | | | | |
| Genotype |  |  |  | |  |  |  |
| Val/Val | 32 (22.4) | 20 (40.0) | Baseline | | | Baseline | |
| Val/Met | 78 (54.6) | 23 (46.0) | 0.47 (0.23-0.98) | | 0.04** | 0.33 (0.14-0.74) | 0.01** |
| Met/Met | 33 (23.1) | 7 (14.0) | 0.34 (0.13-0.91) | | 0.03** | 0.24 (0.07-0.75) | 0.01** |
| Verbal ability | | | | | | | |
| Genotype |  |  |  | |  |  |  |
| Val/Val | 38 (24.7) | 14 (35.9) | Baseline | | | Baseline | |
| Val/Met | 83 (53.9) | 18 (46.2) | 0.59 (0.27-1.31) | | 0.19 | 0.59 (0.24-1.49) | 0.27 |
| Met/Met | 33 (21.4) | 7 (18.0) | 0.58 (0.21-1.60) | | 0.29 | 0.51 (0.15-1.74) | 0.28 |
|  |  |  |  | |  |  |  |

* Insufficient covariate data for 1 participant

** p < 0.05

Supplementary Table S2 (continued) Association of BDNF genotype with cognitive decline assuming a general genetic model

| Variable | Impairment | | | Unadjusted analysis | | Adjusted analysis | |
| --- | --- | --- | --- | --- | --- | --- | --- |
|  | Yes, n (%) | No, n (%) | | OR | p-value | OR | p-value |
| Concentration | | | | | | | |
| Genotype |  |  | |  |  |  |  |
| Val/Val | 37 (26.6) | 15 (27.8) | | Baseline | | Baseline | |
| Val/Met | 71 (51.1) | 30 (55.6) | | 1.06 (0.51-2.21) | 0.88 | 0.90 (0.39-2.07) | 0.81 |
| Met/Met | 31 (22.3) | 9 (16.7) | | 0.79 (0.30-2.07) | 0.64 | 0.72 (0.24-2.16) | 0.56 |
| Mental acuity | | | | | | | |
| Genotype |  |  | |  |  |  |  |
| Val/Val | 32 (23.2) | 20 (36.4) | | Baseline | | Baseline | |
| Val/Met | 76 (55.1) | 25 (45.5) | | 0.53 (0.26-1.09) | 0.09 | 0.44 (0.20-1.00) | 0.05 |
| Met/Met | 30 (21.7) | 10 (18.2) | | 0.51 (0.20-1.31) | 0.16 | 0.50 (0.18-1.43) | 0.20 |
| Functional interference | | | | | | | |
| Genotype |  |  | |  |  |  |  |
| Val/Val | 41 (26.3) | 11 (29.7) | | Baseline | | Baseline | |
| Val/Met | 82 (52.6) | 19 (51.4) | | 0.87 (0.38–2.01) | 0.75 | 0.71 (0.27-1.86) | 0.48 |
| Met/Met | 33 (21.2) | 7 (18.9) | | 0.87 (0.30-2.51) | 0.80 | 0.64 (0.18-2.28) | 0.50 |
|  | | |  | | |  | |
| **Objective cognitive function** | | | **(n = 120)** | | | **(n = 119)*** | |
| Response speed | | | | | | | |
| Genotype |  |  | |  |  |  |  |
| Val/Val | 31 (30.1) | 3 (17.7) | | Baseline | | Baseline | |
| Val/Met | 51 (49.5) | 11 (64.7) | | 2.23 (0.58-8.62) | 0.25 | 3.38 (0.75-15.24) | 0.11 |
| Met/Met | 21 (20.4) | 3 (17.7) | | 1.48 (0.27-8.03) | 0.65 | 2.02 (0.30-13.49) | 0.47 |

* Insufficient covariate data for 1 participant

Supplementary Table S2 (continued) Association of BDNF genotype with cognitive decline assuming a general genetic model

| Variable | Impairment | | Unadjusted analysis | | Adjusted analysis | |
| --- | --- | --- | --- | --- | --- | --- |
|  | No, n (%) | Yes, n (%) | OR | p-value | OR | p-value |
| Learning and memory | | | | | | |
| Genotype |  |  |  |  |  |  |
| Val/Val | 30 (28.6) | 4 (26.7) | Baseline | | Baseline | |
| Val/Met | 56 (53.3) | 6 (40.0) | 0.80 (0.21-3.07) | 0.75 | 1.26 (0.26-6.06) | 0.77 |
| Met/Met | 19 (18.1) | 5 (33.3) | 1.97 (0.47-8.29) | 0.35 | 2.78 (0.46-16.89) | 0.27 |
| Working memory | | | | | | |
| Genotype |  |  |  |  |  |  |
| Val/Val | 30 (29.1) | 4 (23.5) | Baseline | | Baseline | |
| Val/Met | 54 (52.4) | 8 (47.1) | 1.11 (0.31-4.00) | 0.87 | 1.10 (0.27-4.42) | 0.89 |
| Met/Met | 19 (18.5) | 5 (29.4) | 1.97 (0.47-8.29) | 0.35 | 2.18 (0.42-11.25) | 0.35 |
| Multitasking | | | | | | |
| Genotype |  |  |  |  |  |  |
| Val/Val | 29 (26.4) | 5 (50.0) | Baseline | | Baseline | |
| Val/Met | 58 (52.7) | 4 (40.0) | 0.40 (0.10-1.60) | 0.20 | 0.31 (0.05-1.81) | 0.17 |
| Met/Met | 23 (20.9) | 1 (10.0) | 0.25 (0.03-2.31) | 0.22 | 0.36 (0.03-4.55) | 0.43 |
| Sustained attention | | | | | | |
| Genotype |  |  |  | | | |
| Val/Val | 29 (31.5) | 5 (17.9) | Baseline | | Baseline | |
| Val/Met | 46 (50.0) | 16 (57.1) | 2.02 (0.67-6.10) | 0.21 | 2.70 (0.76-9.58) | 0.13 |
| Met/Met | 17 (18.5) | 7 (25.0) | 2.39 (0.65-8.71) | 0.19 | 4.49 (0.98-20.7) | 0.05 |
|  |  |  |  |  |  |  |

Supplementary Table S3 Univariate logistic regression examining association of demographic and clinical characteristics with subjective and objective cognitive decline

| Covariate | Subjective (n = 193) | | Objective (n = 120) | |
| --- | --- | --- | --- | --- |
|  | OR (95% CI) | p-value | OR (95% CI) | p-value |
| Age in years | 0.98 (0.95-1.02) | 0.28 | 1.03 (0.99-1.08) | 0.14 |
| Ethnicity |  | 0.48 |  | 0.79 |
| Chinese | Reference |  | Reference |  |
| Malay | 2.18 (0.83-5.72) |  | 1.73 (0.46-6.53) |  |
| Indian | 1.08 (0.32-3.68) |  | 1.16 (0.22-6.01) |  |
| Others | 0.97 (0.18-5.18) |  | 2.89 (0.53-15.62) |  |
| Education level** |  | 0.52 |  | 0.25 |
| Primary school | Reference |  | Reference |  |
| Secondary school | 0.70 (0.29-1.68) |  | 0.33 (0.08-1.37) |  |
| Pre-university | 0.97 (0.35-2.67) |  | 0.24 (0.05-1.12) |  |
| Graduate | 0.51 (0.18-1.46) |  | 0.27 (0.06-1.21) |  |
| Working status** |  | 0.56 |  | 0.90 |
| Yes | Reference |  | Reference |  |
| No | 1.25 (0.67-2.33) |  | 0.95 (0.44-2.04) |  |
| Menopausal status |  | 0.32 |  | 0.15 |
| Premenopausal | Reference |  | Reference |  |
| Postmenopausal | 0.71 (0.39-1.32) |  | 1.71 (0.83-3.54) |  |
| Cancer staging |  | 0.98 |  | 0.30 |
| Stage I | Reference |  | Reference |  |
| Stage II | 1.09 (0.44-2.70) |  | 1.21 (0.44-3.33) |  |
| Stage III | 1.06 (0.36-3.08) |  | 2.41 (0.68-8.47) |  |
| Radiotherapy |  | 0.16 |  | 0.51 |
| Yes | Reference |  | Reference |  |
| No | 0.62 (0.32-1.22) |  | 0.77 (0.36-1.67) |  |
| Surgery |  | 0.06 |  | 0.43 |
| Lumpectomy | Reference |  | Reference |  |
| Mastectomy | 0.55 (0.29-1.02) |  | 1.34 (0.64-2.81) |  |
| Chemotherapy |  | 0.19 |  | 0.66 |
| Anthracycline-based | Reference |  | Reference |  |
| Non anthracycline-based | 0.64 (0.33-1.23) |  | 0.85 (0.40-1.79) |  |
| Baseline BAI score | 1.05 (1.01-1.10) | 0.02* | 0.97 (0.93-1.02) | 0.27 |
| Baseline BFI score | 1.26 (1.08-1.48) | 0.004* | 0.95 (0.79-1.14) | 0.58 |
| Baseline EORTC-SL score | 1.01 (1.00-1.02) | 0.13 | 1.00 (0.99-1.01) | 0.79 |

* p < 0.05

** Data not available for 1 subject
